# Association between Serum Varicella-Zoster Virus IgM and IgG and Prognosis of Ramsay Hunt Syndrome

**DOI:** 10.3390/jcm12155164

**Published:** 2023-08-07

**Authors:** Seok Hwan Chung, Jung Min Kim, Hwa Sung Rim, Seung Geun Yeo, Sang Hoon Kim

**Affiliations:** Department of Otorhinolaryngology, Head and Neck Surgery, Kyung Hee University School of Medicine, Kyung Hee University Medical Center, Seoul 02447, Republic of Korea; seokhwanchung@naver.com (S.H.C.); kpax1727@naver.com (J.M.K.); marslover@naver.com (H.S.R.); yeo2park@gmail.com (S.G.Y.)

**Keywords:** Ramsay Hunt syndrome, facial palsy, varicella-zoster virus, serum immunoglobulin

## Abstract

Ramsay Hunt syndrome (RHS) has a poor prognosis because of varicella-zoster virus (VZV) infection. This is most closely related to severe inflammation in the geniculate ganglion of the facial nerve due to VZV infection or reactivation. This study investigated whether there were differences in the prognosis and accompanying symptoms of facial paralysis based on the presence or absence of VZV IgM and IgG antibodies. This study was conducted as a retrospective chart analysis of 105 patients with RHS who were admitted to our hospital between 2015 and 2021. The House–Brackmann (HB) grade and electroneurography (ENoG) was used to evaluate the degree of facial paralysis. Patients’ subjective symptoms were evaluated by dividing them into dizziness, tinnitus, hyperacusis, and hearing loss. No difference was observed in the initial HB grade with or without IgM; however, the final HB grade was significantly higher in IgM-positive patients than in IgM-negative patients (*p* < 0.05). Further, when IgM was positive, the value of the orbicularis oculi muscle in the ENoG test results was significantly higher (*p* < 0.05), and symptoms of tinnitus and hyperacusis occurred more frequently (*p* < 0.05). The initial and final HB grades were significantly higher in IgG-positive patients than in IgG-negative patients (*p* < 0.05). When IgG was positive, the values of nasalis and oris muscles in the ENoG test results were significantly higher (*p* < 0.05), and symptoms of dizziness occurred more frequently (*p* < 0.05). This study confirmed that the more active the immunological action of the VZV in the body, the greater the damage to the facial and vestibulocochlear nerves, which are associated with the degree of facial paralysis and the accompanying otologic symptoms.

## 1. Introduction

Varicella-zoster virus (VZV), also known as the chickenpox–shingles virus, is one of the most common causes of infection in the central nervous system. VZV infection can lead to encephalitis, meningitis, Ramsay Hunt syndrome (RHS), stroke, and other neurological complications. In particular, when VZV infects the seventh cranial nerve, which controls the facial muscles, it can cause peripheral facial paralysis along with symptoms such as tinnitus, pain and blisters around the ear, and dizziness. Despite its infrequency, RHS presents a significant challenge for clinicians due to its potential for severe complications and the need for early diagnosis and prompt management [1]. RHS accounts for approximately 6.1% of all facial paralysis cases, and its prognosis is worse than that of Bell’s palsy [2]. This is thought to be due to the close association between the VZV infection and reactivation of preexisting VZV in the geniculate ganglion of the facial nerve, which leads to severe inflammation [3].

In addition to serological tests that use enzyme-linked immunosorbent assay (ELISA) to detect VZV infection, polymerase chain reaction has recently been introduced as a method for detecting VZV infection in saliva, blood, or nervous tissue samples, and several studies have reported a relationship between VZV infection and RHS [4,5,6].

Specifically, in patients with RHS, VZV DNA was found not only in the external ear and oral vesicle, but also in the middle-ear mucosa, facial nerve sheath, and cerebrospinal fluid [5]. Furthermore, according to another previous study, the higher the VZV DNA titer collected from the saliva of patients with RHS, the poorer the prognosis of facial paralysis after the end of treatment [6]. In addition, when RHS occurred due to a new infection or reactivation of VZV, the higher the VZV DNA titer in the body, the higher the incidence of vestibulocochlear symptoms due to the progression of neuritis or labyrinthitis [4]. These study results indicate that there is a close relationship between RHS and VZV infection, and it can be confirmed that the higher the activity of VZV present in the body, the higher the severity of the disease and the worse the prognosis.

This study aimed to investigate whether there were differences in the prognosis of facial paralysis and accompanying symptoms based on the presence or absence of VZV IgM and IgG antibodies, which are responsible for the body’s immune response to VZV. To our knowledge, no previous research has been conducted on this topic, and we believe that this study will provide insights into predicting the prognosis and symptoms of facial paralysis in RHS patients.

## 2. Materials and Methods

### 2.1. Patients

In this study, we retrospectively analyzed the medical records of 105 patients with RHS who were admitted to the Department of Otorhinolaryngology at Kyung Hee University Hospital (Seoul, South Korea) and underwent high-dose steroid therapy from 2015 to 2022 (8 years). The diagnostic criteria for RHS were as follows: (1) acute or subacute peripheral facial palsy; (2) vesicular rash in the pattern of the external ear, external auditory canal, or oropharynx; and (3) absence of any brainstem or cerebellar lesions on magnetic resonance imaging [7]. Patients with facial palsy caused by a tumor in the brain, trauma, or inflammatory ear diseases were excluded from the study. Patients who were admitted to the hospital within seven days of the onset of RHS and completed high-dose steroid and antiviral treatments were included in the study. All the patients were examined and treated according to the same protocol. The patients’ medical records included information on sex, age, medical history, accompanying neurological symptoms, and the direction and severity of facial palsy. Their medical history included the presence of diabetes, hypertension, and accompanying neurological symptoms, including dizziness, tinnitus, and hearing loss, which were obtained through a medical interview at admission.

We used the House–Brackmann (HB) grading system to assess the degree of facial palsy at admission and six months after onset [8]. The HB grading system is divided into six stages: stage I, normal; stage II, mild dysfunction (with some weakness observed on close inspection and mild synkinesis); stages III and IV, moderate facial palsy; and stages V and VI, complete facial palsy (with significant facial asymmetry even when the muscles are maximally contracted) [8]. Patients with an HB grade of II–IV on admission were classified as having incomplete facial palsy, whereas those with an HB grade of V or VI were classified as having complete facial palsy. Patients with a final HB grade of I or II after high-dose steroid therapy were classified as having a satisfactory recovery, whereas those with an HB grade of III–V were classified as having a nonsatisfactory recovery.

High-dose steroids were administered to patients who met the inclusion criteria. High-dose steroid therapy involved the use of 5 mg of steroids per kg of body weight for the first 4 days, followed by a reduction over 6–8 days (80 mg/day for 4 days, 60 mg/day for 2 days, 40 mg/day for 2 days, 20 mg/day for 2 days, and 10 mg/day for 2 days). High-dose steroids were used with caution even in normal patients without underlying diseases because they can cause metabolic effects and an increased vascular pressure as well as worsen underlying conditions such as hypertension or diabetes. In particular, for patients with diabetes whose blood sugar control was poor, the endocrinology department was consulted, and they recommended using oral antidiabetic drugs or insulin during the high-dose steroid therapy. Moreover, for patients with hypertension, when blood pressure was not continuously controlled while taking oral corticosteroids, an oral antihypertensive agent was additionally taken by referring to the cardiology department. Additionally, all patients were administered famciclovir 750 mg orally once daily for one week, and the use of eye drops, ointments, and eye patches during sleep was recommended to protect the cornea [9].

### 2.2. Detection of Serum VZV IgM and IgG Antibodies

Blood samples were collected from the patients upon admission and stored in a freezer at −20 °C. VZV IgM and IgG antibodies were detected using ELISA. IgM antibodies were measured using a solid-phase enzyme immunoassay (EIA), and IgG antibodies were measured using the captured EIA. Results were classified as negative when the values were <0.90, intermediate when between 0.90 and 1.10, and positive when >1.10. Patients with intermediate results were excluded from the study [10]. Our study did not include the specific quantities of VZV IgM and IgG antibodies as they were not provided by our hospital.

### 2.3. Electroneurography

To accurately evaluate the axonal degeneration of the facial nerve, the prognosis of facial paralysis, electroneurography (ENoG) was performed between 5 and 14 days after the onset of RHS [11,12]. The test was first conducted on facial areas without paralysis and then on areas with paralysis, evaluating the function of the frontalis, orbicularis oculi, nasalis, and orbicularis oris muscles by positioning bipolar nerve stimulators on the temporal, zygomatic, buccal, marginal mandibular, and cervical branches of the facial nerve [4,13].

For measurement, surface electrodes were attached to the upper and lower lateral sides of the eyebrow, nasolabial fold, and upper lip, and the ground electrode was attached to both wrists. The nerve stimulator was positioned at the stylomastoid foramen and started with a low current of 10 mA, gradually increasing the intensity of stimulation by 10 mA up to a maximum current of 80 mA. The maximum electrical stimulation applied to the muscle was 1 Hz AC for 0.2 ms, and all tests were conducted using the DS7A model (Welwyn Garden City, Hertfordshire, UK) [14,15]. The maximum response to nerve stimulation on the side with facial paralysis was divided by the maximum response to nerve stimulation on the side without facial paralysis to calculate the ratio.

### 2.4. Statistical Analysis

For categorical data, such as sex, direction of facial paralysis, HB grade, medical history, and symptoms reported by the patients, chi-square and multiple logistic regression tests were used. For the normality test for continuous data, the Kolmogorov–Smirnov test was conducted, such as for the ENoG results. Then, to analyze the difference between the mean, an independent *t*-test was used. Multiple regression tests were used to examine the impact of VZV IgM and IgG on each dependent variable, with adjustments for sex and age. SPSS statistical software was used for the statistical analysis, with a significance level of *p* < 0.05.

## 3. Results

Of the 105 patients with RHS, 38 were IgM-positive and 71 were IgM-negative, whereas 91 were IgG-positive and 18 were IgG-negative. The results are summarized in Table 1. The mean age of IgM-positive patients was 50.55 ± 19.03 years, while that of IgM-negative patients was 51.35 ± 14.43 years, with no statistically significant difference between the two groups. IgM-positive patients included 22 men and 16 women, while IgM-negative patients included 16 men and 55 women. This resulted in a statistically significant difference in sex between the two groups based on IgM positivity. However, this was attributed to the differences in data collection. In the IgM-positive group, 5 had hypertension and 1 had diabetes, while in the IgM-negative group, 11 had hypertension and 9 had diabetes, indicating no statistically significant difference between the two groups. In addition, among IgM-positive patients, 26 had dizziness, 14 had tinnitus, 21 had hyperacusis, and 11 had hearing loss; among IgM-negative patients, 37 had dizziness, 15 had tinnitus, 30 had hyperacusis, and 17 had hearing loss. No statistically significant differences were observed in coexisting symptoms between the IgM-positive and -negative groups.

The mean age of IgG-positive patients was 50.73 ± 15.80 years, while that of IgG-negative patients was 52.77 ± 17.87 years, indicating no statistically significant difference between the two groups. In the IgG-positive group, 35 were men and 56 were women, while in the IgG-negative group, 3 were men and 15 were women. No significant difference was observed in terms of sex between the two groups. In the IgG-positive group, 16 patients had hypertension and 10 had diabetes, whereas there were no patients with hypertension or diabetes in the IgG-negative group, indicating no statistically significant difference between the two groups. In addition, among IgG-positive patients, 49 had dizziness, 26 had tinnitus, 39 had hyperacusis, and 21 had hearing loss; among IgG-negative patients, 14 had dizziness, 3 had tinnitus, 12 had hyperacusis, and 7 had hearing loss. No statistically significant differences were observed in coexisting symptoms between the IgG-positive and -negative groups (Table 1).

Table 2 shows the initial and final HB grades based on the IgM level. No statistically significant difference was observed between the two groups in terms of the initial HB grade. However, a statistically significant difference was observed in the prognosis of facial paralysis between the IgM-positive and -negative groups based on the final HB grade (initial HB grade, *p* = 0.553; final HB grade, *p* < 0.0001) (Table 2). Next, we examined the initial and final HB grades based on IgG levels.

**Figure 1:**
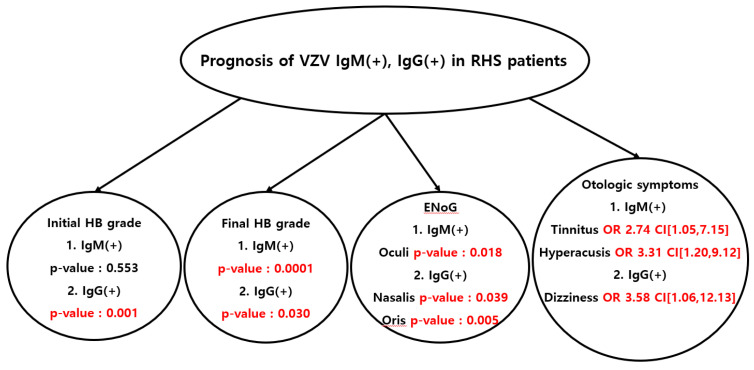
Prognosis of VZV IgM(+), IgG(+) in Ramsay Hunt syndrome patients.

The initial and final HB grades based on IgG levels are shown in Table 3. Both the initial and final HB grades were significantly higher in the IgG-positive group than in the IgG-negative group (initial HB grade, *p* = 0.001; final HB grade, *p* = 0.030). The Kolmogorov–Smirnov test was performed to test the normality of the ENoG result values. As a result of the analysis, it was confirmed that the value of the ENoG satisfied normality (*p* > 0.05). The ENoG results for the four facial muscles based on the IgM and IgG levels are shown in Table 4. The mean values of the ENoG results for the four facial muscles were higher in the IgM-positive group than in the IgM-negative group, indicating that more nerve degeneration had occurred. In particular, the ENoG result for the orbicularis oculi muscle was significantly higher (*p* = 0.018). Similarly, the mean ENoG values for the four facial muscles were higher in the IgG-positive group than in the IgG-negative group. The average ENoG values for nasalis, orbicularis oris, and the four facial muscles were significantly higher in the IgG-positive group than in the IgG-negative group (nasalis: *p* = 0.039, orbicularis oris: *p* = 0.005, average: *p* = 0.036). To evaluate the effects of IgM and IgG on neurological symptoms, a multiple logistic regression analysis was performed (Table 5). In the multiple logistic regression analysis adjusted for sex and age, the IgM-positive group had more symptoms of tinnitus and aural fullness than the IgM-negative group, whereas the IgG-positive group had more symptoms of dizziness than the IgG-negative group. All the above analyses are summarized as follows (Figure 1).

## 4. Discussion

In this study, we confirmed the correlation between VZV IgM and IgG antibodies collected from the blood of RHS patients and the prognosis of facial paralysis and related symptoms. Based on the results of the initial and final HB grading systems and ENoG, we concluded that the higher the activity of VZV IgM and IgG, which play an important immunological role in VZV, the worse the prognosis of facial paralysis. In addition to facial paralysis symptoms, we also found that the higher the activity of VZV IgM and IgG, the higher the frequency of accompanying symptoms.

In the context of VZV infection, VZV Ig refers to the specific antibodies produced by the immune system in response to the presence of the varicella-zoster virus. These antibodies, including IgM and IgG, play a critical role in detecting and neutralizing the virus, aiding in the diagnosis and management of VZV-related diseases. Several studies have been conducted so far regarding the long-term prognosis of facial paralysis and accompanying symptoms in patients with RHS. According to one previous study, the prognosis of facial paralysis was worse in patients with RHS, who were old, had hypertension or diabetes mellitus as an underlying disease, and had dizziness as a comorbidity [16]. In another previous study, it was reported that the higher the neutrophil-to-lymphocyte ratio and neutrophil count before high-dose steroid treatment in patients with RHS, the poorer the prognosis of facial paralysis. A high neutrophil-to-lymphocyte ratio and neutrophil count mean that the body’s immune response is actively occurring, and through this, it can be confirmed that the inflammatory response to VZV in the facial nerve sheath plays an important role in the development of facial paralysis in RHS. In addition, it was deduced that the more inflammatory reactions occur, the poorer the prognosis of patients with RHS [17]. As such, although many studies have been conducted on the prognostic factors of RHS, studies on VZV IgM and IgG antibodies, which have a direct immune response against VZV, have not been conducted so far.

VZV IgM and IgG are synthesized in the body in response to VZV infection, which causes vesicular rashes on the skin; synthesis begins in the body between two and five days, reaching a maximum level between two and three weeks [18]. VZV IgM is responsible for the acute immune response and accounts for approximately 10% of the total immunoglobulins, which are only found in the blood and lymphatic fluid. It is also synthesized between 6 and 10 days after the occurrence of vesicular rashes on the skin and reaches its maximum level 1 month before gradually decreasing and becoming negative in the body after 10 weeks [19]. According to a study conducted by Min et al., VZV IgM is not a highly specific marker because it is detected only in approximately 37% of patients with VZV infection through ELISA testing. In the present study, the proportion of patients with positive VZV IgM results was 35%, which is consistent with the results of previous studies. Based on these findings, we concluded that not all patients infected with VZV have detectable VZV IgM levels. In addition, 17% of patients with positive VZV IgM become negative within 1 week, 27% between 1 and 2 weeks, 36% between 2 and 4 weeks, and all become negative after 10 weeks [20].

VZV IgG is the most abundant immunoglobulin in the body and is responsible for long-lasting immune effects, comprising approximately 80% of the total immunoglobulin. Additionally, VZV IgG can be distributed throughout the body, not just in the blood and lymphatic fluid. According to Charlotte et al., VZV IgG is expressed in approximately 80% of patients with acute VZV infection [21]. In the present study, VZV IgG positivity in patients was consistent with that reported in a previous study (83%). Furthermore, in cases of VZV reactivation from latency, VZV IgG levels increased up to fourfold, reached a maximum after one month, gradually decreased over the following six months, and then stabilized at a constant level.

When comparing the characteristics and differences between VZV IgM and IgG, a notable feature of this study is that in cases where VZV IgG is positive, the severity and prognosis of facial paralysis are worse than in cases where VZV IgM, which is known to play an important role in acute immune response, is positive. This is because VZV IgM is synthesized faster in the body during the acute phase and reaches its maximum level faster than VZV IgG, which acts over a longer period of six months or more, with a greater difference in immune activity at a ratio of 1:8. Therefore, VZV IgG is more actively involved in immune activity and yields statistically significant results in terms of the initial HB grade and mean ENoG values. However, because the sample size of the study population with RHS was limited to 105 individuals, further research is necessary.

Furthermore, based on our results, there was also a correlation between VZV IgM and IgG and otologic symptoms. When VZV IgM is positive, symptoms of tinnitus and auditory hypersensitivity are relatively more common, whereas when VZV IgG is positive, symptoms of dizziness are more common. This suggests that an increase in VZV IgM and IgG, which act on the vestibulocochlear nerve, is more likely to result in otologic symptoms. However, it is difficult to explain why VZV IgM and IgG cause different otologic symptoms in the positive cases. Further molecular biological research can confirm whether VZV IgM and IgG specifically act on the cochlear and vestibular nerves, respectively, and this topic can be discussed further.

## 5. Conclusions

In this study, we evaluated the effects of VZV IgM and IgG antibodies collected from the blood of patients with RHS on the severity of early facial paralysis, the prognosis of facial paralysis after treatment, and accompanying otologic symptoms. We found that the prognosis of facial paralysis was worse when each serological marker was positive, as confirmed by the HB grade and ENoG results for the four facial muscles. In addition, we found more cases of tinnitus and dizziness in the positive serological marker group than in the negative serological marker group. The results of this study confirmed a correlation between the immunological action of VZV in the body and damage to the facial and vestibulocochlear nerves, which has an effect on the severity of facial paralysis and its accompanying otologic symptoms. As a limitation of this study, when VZV IgM and IgG antibodies were tested at our hospital, the results were only negative or positive, not quantitative values. The higher the quantitative level of VZV IgM and IgG antibodies, the poorer the prognosis of facial paralysis and the more otologic symptoms may occur; thus, additional research is needed. Moreover, our institution conducted tests for VZV IgM and IgG antibodies once at the time of admission, but these results may vary depending on when the patient was infected with the varicella-zoster virus. In addition, VZV IgM and IgG antibodies in the body become negative over time after infection with VZV, which can affect the prognosis of RHS. It is considered a limitation that this study could not conduct such an analysis due to the retrospective nature of the study. In a future study, additional tests for VZV IgM and IgG antibodies need to be performed, not just one test at the time of admission.

## Figures and Tables

**Table 1:** Demographic features of patients with Ramsay Hunt syndrome classified by serum IgM and IgG.

Ramsay Hunt Syndrome						
Parameter	IgM(+) (n = 38)	IgM(−) (n = 71)	*p*-Value	IgG(+) (n = 91)	IgG(−) (n = 18)	*p*-Value
Age (years)	50.55 ± 19.03	51.35 ± 14.43	0.806	50.73 ± 15.80	52.77 ± 17.87	0.433
Sex (n)						
Men	22 (57.89)	16 (22.53)	* 0.001	35 (38.46)	3 (16.67)	0.076
Women	16 (42.11)	55 (77.47)		56 (61.54)	15 (83.33)	
Direction (n)						
Right	14 (36.84)	25 (35.21)	0.886	39 (42.86)	5 (27.78)	0.233
Left	24 (63.16)	46 (64.79)		52 (57.14)	13 (72.22)	
Underlying disease						
Hypertension	5 (13.15)	11 (15.49)	0.743	16 (17.58)	0 (0)	0.054
Diabetes	1 (2.63)	9 (12.68)	0.083	10 (10.98)	0 (0)	0.140
Accompanying symptoms						
Dizziness	26 (68.42)	37 (52.11)	0.100	49 (53.85)	14 (77.78)	0.060
Tinnitus	14 (36.84)	15 (21.12)	0.077	26 (28.57)	3 (16.67)	0.296
Hyperacusis	21 (55.26)	30 (42.25)	0.195	39 (42.86)	12 (66.67)	0.064
Hearing disturbance	11 (0.29)	17 (23.94)	0.569	21 (23.08)	7 (38.89)	0.161

* Indicates statistical signifcance.

**Table 2:** Differences in the initial and final HB grades between serum IgM-negative and -positive groups.

	Initial HB Grade			Final HB Grade		
Parameter	II–III	IV–VI	*p*-Value	I–II	III–V	*p*-Value
IgM(−)	36 (34.52%)	35 (36.48%)	0.553	64 (54.06%)	7 (16.94%)	* <0.0001
IgM(+)	17 (18.48%)	21 (19.52%)		19 (28.94%)	19 (10.89%)	

* Indicates statistical signifcance.

**Table 3:** Differences in the initial and final HB grades between serum IgG-negative and -positive groups.

	Initial HB Grade			Final HB Grade		
Parameter	II–III	IV–VI	*p*-Value	I–II	III–V	*p*-Value
IgG(−)	15 (13.76%)	3 (2.75%)	* 0.001	15 (13.76%)	3 (2.75%)	* 0.030
IgG(+)	38 (34.86%)	53 (48.63%)		51 (46.79%)	40 (36.70%)	

HB, House–Brackmann. * Indicates statistical signifcance.

**Table 4:** Differences in the ENoG values of the four facial muscles between the IgM- and IgG-negative and positive groups.

Parameter	Serum IgM(−)	Serum IgM(+)	*p*-Value	Serum IgG(−)	Serum IgG(+)	*p*-Value
Frontalis	62.18 ± 28.00	71.89 ± 27.89	0.097	55.61 ± 39.60	67.40 ± 25.18	0.105
Oculi	61.33 ± 28.60	74.18 ± 22.20	* 0.018	56.56 ± 32.26	67.65 ± 25.83	0.114
Nasalis	66.07 ± 26.61	71.45 ± 25.05	0.307	56.39 ± 30.08	70.23 ± 24.76	* 0.039
Oris	78.64 ± 23.16	78.53 ± 26.62	0.980	64.06 ± 34.51	81.48 ± 20.80	* 0.005
Average	67.06 ± 25.21	73.93 ± 24.73	0.174	58.15 ± 33.48	71.69 ± 22.72	* 0.036

ENoG, electroneurography. * Indicates statistical signifcance.

**Table 5:** Effects of IgM and IgG on otologic symptoms based on the multiple logistic regression model.

Parameter	Serum IgM		OR	CI		*p*-Value	Serum IgG		OR	CI		*p*-Value
Dizziness	IgM(+)	1.92		0.79	4.67	0.1516	IgG(+)	3.58		1.06	12.13	* 0.0404
	IgM(−)	1.00					IgG(−)	1.00				
Tinnitus	IgM(+)	2.74		1.05	7.15	* 0.0393	IgG(+)	2.16		0.57	8.21	0.2599
	IgM(−)	1.00					IgG(−)	1.00				
Hyperacusis	IgM(+)	3.31		1.20	9.12	* 0.0203	IgG(+)	2.85		0.88	9.23	0.0811
	IgM(−)	1.00					IgG(−)	1.00				
Hearing disturbance	IgM(+)	1.21		0.45	3.21	0.7100	IgG(+)	2.21		0.71	6.87	0.1708
	IgM(−)	1.00					IgG(−)	1.00				

OR, odds ratio; CI, confidence interval. * Indicates statistical signifcance.

## Data Availability

Data were collected during the study at the Kyunghee Medical Center.
